# Production of Antioxidant Transfersomes by a Supercritical CO_2_ Assisted Process for Transdermal Delivery Applications

**DOI:** 10.3390/nano13121812

**Published:** 2023-06-06

**Authors:** Raffaella Squittieri, Lucia Baldino, Ernesto Reverchon

**Affiliations:** 1 Department of Industrial Engineering, University of Salerno, Via Giovanni Paolo II, 132, 84084 Fisciano, SA, Italy; raffaellasquittieri@gmail.com (R.S.); ereverchon@unisa.it (E.R.); 2 Research Center for Biomaterials BIONAM, University of Salerno, Via Giovanni Paolo II, 132, 84084 Fisciano, SA, Italy

**Keywords:** transfersomes, nanovesicles, supercritical CO_2_ process, ascorbic acid, transdermal drug delivery

## Abstract

Transfersomes are deformable vesicles that can transport drugs across difficult-to-permeate barriers in human tissues. In this work, nano-transfersomes were produced for the first time by a supercritical CO_2_ assisted process. Operating at 100 bar and 40 °C, different amounts of phosphatidylcholine (2000 and 3000 mg), kinds of edge activators (Span^®^ 80 and Tween^®^ 80), and phosphatidylcholine to edge activator weight ratio (95:5, 90:10, 80:20) were tested. Formulations prepared using Span^®^ 80 and phosphatidylcholine at an 80:20 weight ratio produced stable transfersomes (−30.4 ± 2.4 mV ζ-potential) that were characterized by a mean diameter of 138 ± 55 nm. A prolonged ascorbic acid release of up to 5 h was recorded when the largest amount of phosphatidylcholine (3000 mg) was used. Moreover, a 96% ascorbic acid encapsulation efficiency and a quasi-100% DPPH radical scavenging activity of transfersomes were measured after supercritical processing.

## 1. Introduction

The skin is the largest organ of the human body and accomplishes multiple defensive and regulatory functions [1]. It is a barrier against external chemical, mechanical, physical, and microbial stresses, resulting in protection against pathogens and water loss. However, skin may be affected by many disorders (e.g., rashes), infections (i.e., viral, bacterial, fungal, and parasitic), injuries caused by cuts or burns, and tumors.

The treatment of skin disorders can be performed by topical applications of drugs on the target site [2]. However, the major challenge in developing this kind of drug delivery system is the transport of the active pharmaceutical ingredient across the barrier of the skin [3]. Indeed, the skin is formed by three main layers, starting from the outer one: the epidermis, dermis, and hypodermis. The external layer of the skin, named stratum corneum, is constituted of a broad 10–15 μm size matrix of keratolytic cells (corneocytes) and represents the main barrier to the topical or transdermal delivery of cosmetics and therapeutic drugs [4].

Liposomes and niosomes are among the most studied vesicular systems for drug delivery applications. However, they are accompanied by limitations when used as topical or transdermal drug carriers: due to their rigid nature, liposomes and niosomes are trapped on the stratum corneum layer, causing reduced penetration of the drug into the deeper cutaneous tissues.

To overcome this skin barrier, a new generation of liposomes, called transfersomes, has been developed [3,5]. Their name means “carrying body” [4] and, structurally, they are formed by an aqueous core surrounded by a lipid bilayer [5]. The typical characteristic of these vesicles is determined by the addition of edge activators to phospholipids in the lipid bilayer. Edge activators are single-chain surfactants (sodium deoxycholate, spans, and tweens), and their insertion between the phospholipids can generate discontinuities in the structure, producing ultra-deformable vesicles [2,6]. Thanks to their ultra-deformable nature, transfersomes can cross the stratum corneum barrier of the skin and penetrate through orifices that are five to ten times smaller than their diameter [5].

The conventional methods used for transfersome production are the thin film hydration technique, reverse-phase evaporation, ethanol injection, vortex-sonication, and mirofluidization [4]. Qushawy et al. [7] produced transfersomes loaded with miconazole nitrate for the treatment of superficial fungal infections using the thin lipid film hydration technique followed by sonication to reduce the vesicle size. The optimal formulation was obtained using Span80 as an edge activator, 300 mg of phospholipids, and a lipids-to-edge activator ratio of 90:10. Transfersomes showed a mean diameter of 71 nm and a drug encapsulation efficiency of 86%. However, the sonication process, selected to reduce the vesicle dimension, is difficult to scale up. Using the same production method, Chen et al. [8] developed carvedilol loaded transfersomes for skin-targeted delivery. The optimal formulation produced vesicles of about 116 nm with an encapsulation efficiency of around 94%, but ζ-potential was 11 mV. Dalimunthe et al. [9] prepared transfersomes loaded with diclofenac using the vortex-sonication method. Formulations prepared using phosphatidylcholine and Tween80 at an 85:15 ratio produced transfersomes with the highest encapsulation efficiency (94%), but they were characterized by a mean diameter of 617 nm. Salim et al. [10] studied rifampicin and vancomycin co-loaded in transfersomes for the treatment of cutaneous leishmaniasis. These transfersomes were produced by the ethanol injection method, testing different lipids, surfactant ratios, agitation speeds, and injection rates. The optimized transfersomes showed a mean size of 168 nm, a low PDI (0.183), and an encapsulation efficiency of 58% for rifampicin and 86% for vancomycin. Vasanth et al. [3] formulated topically applicable adapalene-loaded transfersome gels with the synergistic effect of vitamin C for the treatment of acne vulgaris. Adapalene-loaded transfersomes were produced by reverse-phase evaporation using soya lecithin, cholesterol, Tween80, and sodium deoxycholate in different ratios. Transfersomes showed a mean size in the range of 280 to 400 nm, PDI values between 0.416 and 0.800, ζ-potential between −38 and −20 mV, and an encapsulation efficiency from 32 to 70%. In summary, all these production techniques suffer from some limitations, as they are intrinsically batch and time-consuming processes [11,12], use organic solvents [13], do not assure a control on the size and shape of the vesicles [14], and generally lead to low drug encapsulation efficiency.

A continuous supercritical CO_2_ (SC-CO_2_) assisted process, named SuperSomes [15], has been proposed for vesicle production with the aim of overcoming the previously described limits. Using a versatile lab-scale plant, liposomes and niosomes of nanometric size, characterized by high encapsulation efficiencies (greater than 85%) of different drugs [15,16], have been obtained.

Therefore, the aim of this work is the production the first time production of transfersomes by using SuperSomes. Phosphatidylcholine was selected as the lipid fraction, and Tween80 and Span80 were selected as edge activators. Several transfersomal formulations were tested to investigate the impact of the kind of edge activator, phospholipid to edge activator weight ratio, and phospholipid amount on the physicochemical properties of vesicles, including mean hydrodynamic diameter (MHD), polydispersity index (PDI), and ζ-potential. Then, transfersomes were loaded with ascorbic acid, a model antioxidant compound that is frequently used in topical formulations thanks to its ability to inhibit skin aging by promoting the production of collagen [17]. To validate this system, the deformability, encapsulation efficiency, release profile, and antioxidant activity of the loaded vesicles were studied.

## 2. Materials and Methods

### 2.1. Materials

L-α-Phosphatidylcholine from egg yolk (purity ≥99%), Span^®^ 80 (M_w_ = 428.60 g/mol), Tween^®^ 80 (M_w_ = 1310 g/mol), and L-ascorbic acid powder (M_w_ = 176.12 g/mol, purity ≥99%) were purchased from Merck (Darmstadt, Germany). Ethanol (anhydrous, purity ≥99.9%) was purchased from Carlo Erba Reagents (Cornaredo (MI), Italy). Distilled water was produced in laboratory, using a homemade lab-scale distiller. Carbon dioxide (purity >99.4%) was purchased from Morlando Group Srl (Naples, Italy).

### 2.2. Transfersomes Formulation and SuperSomes Description

A 100 mL volume of ethanolic solution containing phospholipid and surfactant was prepared by magnetic stirring at 250 rpm for 1 h at room temperature. Ascorbic acid was dissolved in 200 mL of water, at a 0.5 mg/mL concentration; the solution was stirred at 250 rpm for 1 h at room temperature. In Table 1, the composition of each formulation is reported.

SuperSomes is a high-pressure plant mainly formed by a static mixer (internal volume 0.15 dm^3^), filled with a stainless-steel packing (Pro-Pak^®^, 0.16 in.^2^, 94% void space, Merck, Darmstadt, Germany), and a vesicle formation vessel (internal volume 500 dm^3^). In the static mixer, CO_2_ is pumped using an Ecoflow^®^ pump (mod. LDC-M-2, Lewa, Leonberg, Germany), and the ethanolic solution is pumped using a Gilson pump (mod. 305, Villiers Le Bel, France). The contact between the ethanolic solution of lipids and SC-CO_2_ promotes the formation of a gas-expanded liquid [15,16,18,19,20] that is delivered through a capillary tube (8 cm length, 1/8 in. external diameter, 0.028 in. wall thickness) inside the formation vessel. The water solution is pumped using a Gilson pump (mod. 305, Villiers Le Bel, France) to the formation vessel and is atomized through a nozzle of 80 μm internal diameter. At the end of the experiment, the system is slowly depressurized, and the mixture of ethanol + CO_2_ is removed by using a separator downstream of the formation vessel, operating at 25 °C and 10 bar. The transfersome suspension is collected in a reservoir located at the bottom of the formation vessel that can be withdrawn using an on/off valve. Temperature along the plant is measured by type J thermocouples (Watlow, Corsico (MI), Italy) and controlled using PID controllers (Series 93, Watlow, Corsico (MI), Italy), whereas pressure is measured by pressure gauges (mod. MP1, OMET, Lecco, Italy). Further details on SuperSomes configuration can be found in [15,16,18,19].

### 2.3. Transfersomes Characterization Techniques

Transfersome suspension was characterized in terms of MHD, PDI, and ζ-potential by dynamic light scattering (DLS, mod. Zetasizer Nano S, Worcestershire, United Kingdom) [21]. All measurements were performed in triplicate.

The morphology of transfersomes was observed by a field emission scanning electron microscope (SEM, mod. LEO 1525, Carl Zeiss SMT AG, Oberkochen, Germany). Before performing this analysis, some drops of transfersomal suspension were deposited on an aluminum stub that was left to dry at room temperature for 2 days. Then, transfersomes were covered by a thin gold layer using a sputter coater (mod. 108 A, Agar Auto Sputter Coater, Stansted, United Kingdom) at 40 mA for 100 s.

A deformability test of ascorbic acid loaded transfersomes was carried out using a MF-Millipore^TM^ 0.1 µm membrane filter (Merck, Darmstadt, Germany). A vacuum pump induced the flux of the aqueous suspension of transfersomes through the porous membrane in a bottle. The size distribution of the transfersomes collected in the bottle after the test was measured by DLS, and the ratio between the largest final vesicle diameter read on the size distribution curve and the pore size of the membrane (0.1 µm) selected for the test represented the deformability factor.

Ascorbic acid encapsulation efficiency (EE%) into transfersomes was measured by an UV-Vis spectrophotometer (mod. Cary 60 UV-Vis, Agilent Technologies, Santa Clara, CA, USA), following the method described in [15]. The ascorbic acid absorbance was read at λ = 260 nm, and EE% was calculated using Equation (1) [16,18,19]:

EE% = (1 − (supernatant concentration)/(theoretical concentration)) × 100(1)

where the supernatant concentration was the unencapsulated drug in the final suspension and the theoretical concentration was the starting concentration of the drug dissolved in water.

To perform drug release tests, 5 mL of transfersome suspension were inserted in a 14,000 Da cut-off dialysis sack (Merck, Darmstadt, Germany) and, then, the sack was immersed in 200 mL of phosphate buffered saline (PBS) at pH 7.4 and 37 °C, stirred at 250 rpm. The ascorbic acid release rate from transfersomes was measured in the dark at λ = 260 nm, using the UV-Vis spectrophotometer previously described.

The DPPH radical scavenging assay was performed using the method described by Chaves et al. [18]: 1 mL of transfersome suspension was gently mixed with 3 mL of fresh DPPH 40 ppm solution and conditioned in the absence of light for 1 h. The values of absorbance were obtained at λ = 517 nm by UV-Vis spectrophotometry. The percentage of DPPH-scavenging activity was calculated using Equation (2):

DPPHscav(%) = (1 − (A_S_ − A_C_)/A_K_) × 100 (2)

where A_S_ is the absorbance of 1 mL of transfersome suspension and 3 mL of DPPH solution, A_C_ is the absorbance of 1 mL of transfersome suspension and 3 mL of ethanol, and A_K_ is the absorbance of 1 mL of ethanol and 3 mL of DPPH solution.

## 3. Results and Discussion

The experiments were carried out while maintaining constant the following operating parameters, since they were optimized in previous studies on the production of other types of nano-vesicles (liposomes and niosomes) using SuperSomes [15,16,18,19]: pressure 100 bar, temperature 40 °C, CO_2_ flow rate 6.5 g/min, ethanolic solution flow rate 3.5 mL/min, water solution flow rate 7 mL/min.

### 3.1. Production of Empty Transfersomes: Effect of the Edge Activator

The first set of experiments was performed to investigate the influence of the kind of edge activator and its concentration on the size, ζ-potential, and stability of transfersomes.

**Figure 1:**
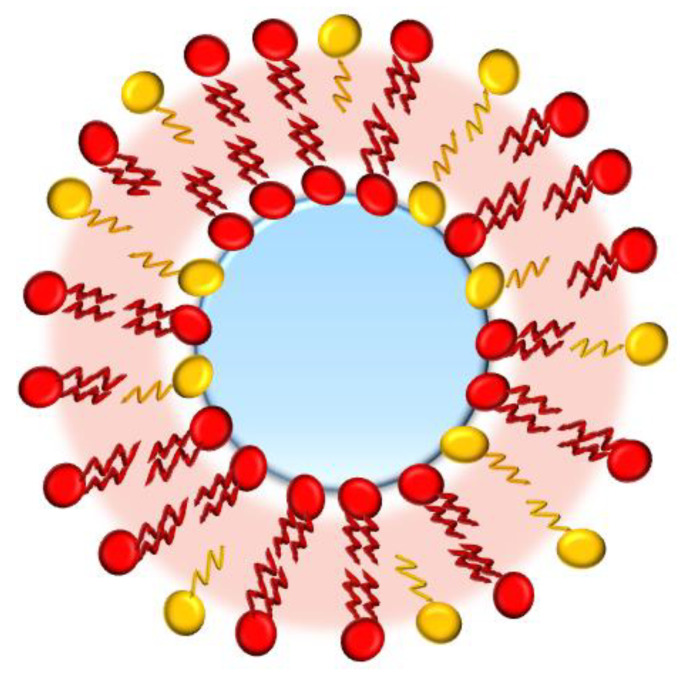
Schematic representation of the transfersome structure (phospholipid in red and edge activator in yellow).

As suggested by the literature [4], Tween80 and Span80 are the most frequently used edge activators for transfersome production, since their chain lengths can interlock more easily inside the phospholipid bilayer, as illustrated in Figure 1.

Experiments from T01 to T06 were performed according to Table 1, in which the amount of phosphatidylcholine was fixed at 2000 mg; whereas two types of edge activators (Tween80 and Span80) and different phosphatidylcholine to edge activator weight ratios (95:5, 90:10, 80:20) were used.

**Figure 2:**
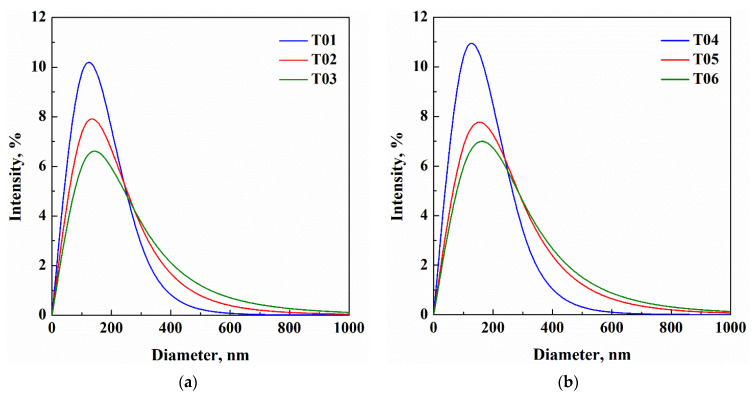
Transfersome size distribution of the samples from T01 to T06, prepared by different edge activators: (**a**) Tween80 and (**b**) Span80.

DLS results are summarized in Table 2, and vesicle size distributions are reported in Figure 2a,b.

**Figure 3:**
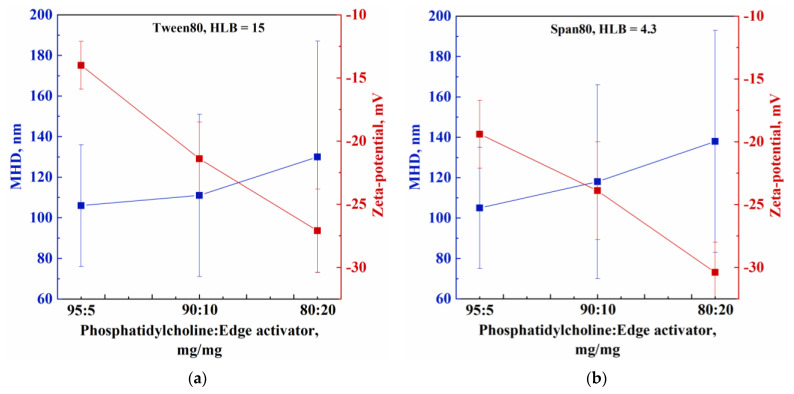
Trend of MHD and Zeta-potential with the phosphatidylcholine to edge activator weight ratio for: (**a**) Tween80 and (**b**) Span80.

In all these experiments, nanometric vesicles were obtained, with a mean diameter ranging from 106 to 130 nm and from 105 to 138 nm, when the edge activator was Tween80 and Span80, respectively. It is worth noting that, regardless of the edge activator used, vesicles size increased when the phospholipid to edge activator weight ratio decreased from 95:5 to 80:20. These results are in agreement with the scientific literature; for example, Bnyan et al. [22] attributed this behaviour to both the larger number of discontinuities due to the interposition of the surfactant molecules in the phospholipid bilayer and the molecular repulsion that can occur between the surfactant and phospholipid molecules. However, in the experiments performed in this work, the size increase was not particularly relevant. As the content of edge activator increased, the value of ζ-potential also increased (absolute value) from −14.1 to −27.1 mV, in the case of Tween80, and from −19.4 to −30.4 mV, in the case of Span80. These trends are summarized in Figure 3a,b for Tween80 and Span80, respectively, in order to highlight the differences depending on the kind of edge activator used. PDI was lower than 0.300 for all samples, except for T03 (0.361); this result indicated a homogenous population of phospholipid vesicles that was suitable for drug delivery applications [23,24]. Vesicles size distributions shown in Figure 2a,b confirm that as the phospholipid to edge activator ratio decreased, transfersomes increased in size and the vesicle size distribution became broader.

To understand the effect of the kind of edge activator, transfersomes produced using different edge activators at the same amount were compared. When the phospholipid to edge activator weight ratio was 95:5, regardless of the kind of edge activator used, no significant differences in the size of the vesicles were detected, whereas, for the other weight ratios, transfersomes containing Span80 were slightly larger than transfersomes containing Tween80 (see Table 2 and Figure 3a,b). As reported in the literature [25], this result is related to the HLB value of the surfactants; i.e., surfactants with higher HLB values, such as Tween80 (HLB = 15), are characterized by a larger interaction with the aqueous phase of the vesicle than surfactants at lower HLB values, such as Span80 (HLB = 4.3); thus, favoring the production of transfersomes with a smaller size. Moreover, transfersomal formulations prepared using Span80 showed higher ζ-potential values (absolute value) than the formulations prepared using Tween80, with a maximum value of −30.4 mV, in line with the literature findings [25]. In particular, Khan et al. [25] tested three types of phospholipids (soya phosphatidylcholine, dimyristoyl phosphatidylcholine, and hydrogenated soya phosphatidylcholine), and Span80 or Tween80 as edge activator, and demonstrated that the formulations prepared using Span80 were characterized by a higher negative charge than those prepared using Tween80.

**Figure 4:**
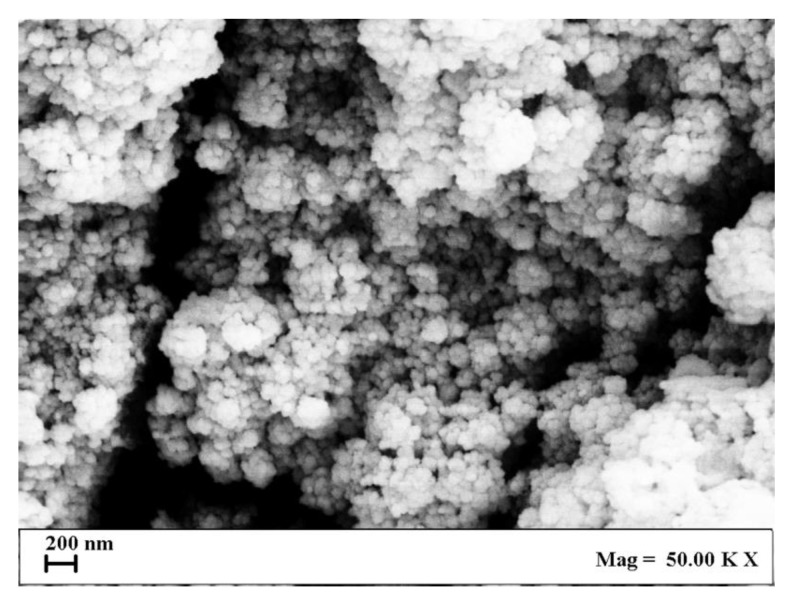
SEM image of T02 vesicles produced by SuperSomes.

Transfersome morphology was observed by SEM, and, in all cases, spherical vesicles with a nanometric size were produced by SuperSomes, in agreement with DLS results. The typical morphology of these vesicles is reported in Figure 4, where a SEM image of the T02 sample is shown.

T01 to T06 samples were analysed again by DLS after 15, 30, and 90 days of storage at 4 °C, to study their stability in terms of variation of MHD, PDI, and ζ-potential, over time. As reported in Table 3, not all samples were stable. Indeed, the T01 sample was stable up to 30 days after production; then, after 90 days, a bimodal distribution of the mean diameter was measured. The T02 sample, after 90 days, showed a significant increase in the PDI value (from 0.267 to 0.518) and a decrease (absolute value) of ζ-potential from −21.4 to −17.9 mV. T03 sample, after 15 days of production, was characterized by a bimodal distribution of the mean diameter. On the other hand, in the case of T04, T05, and T06 samples, no significant changes in the values of MHD, PDI, and ζ-potential, up to 90 days after production, were detected. Therefore, these results suggested that Span80, used as an edge activator, favoured the obtainment of more stable vesicles over time than Tween80.

### 3.2. Production of Empty Transfersomes: Effect of the Phosphatidylcholine Amount

In the second set of experiments, the effect of the amount of phosphatidylcholine on transfersomes physicochemical properties was studied, selecting the formulations prepared using Span80 since they showed a longer stability over time (Table 3). Therefore, Span80 was used as edge activator at the same phosphatidylcholine to edge activator weight ratios as in the previous experiments (95:5, 90:10, 80:20); but with an increase in the amount of phosphatidylcholine from 2000 mg to 3000 mg.

The results of these experiments are summarized in Table 2 (from T07 to T09) and confirm the trend observed in the first set of runs: when the surfactant content increased, the mean diameter of the vesicles increased as well. In particular, vesicles characterized by a mean diameter ranging from 126 to 164 nm, passing from 95:5 to 80:20 weight ratio, were obtained. As in the previous set of experiments, the higher ζ-potential value (absolute value) was obtained for a phosphatidylcholine to edge activator weight ratio equal to 80:20.

Summarizing these results, the increase in the phosphatidylcholine amount produced an increase in the mean diameter of the vesicles of about 20% for each phosphatidylcholine to edge activator weight ratio tested in the two set of experiments. An increase in the PDI value was also observed, in agreement with the results obtained by Salim et al. [10] and Khan et al. [26] that showed a direct proportionality between phosphatidylcholine concentration and PDI. On the other hand, ζ-potential decreased (absolute value) when the quantity of phosphatidylcholine increased. According to Taymouri et al. [27], this effect might be attributed to the exposure of the N-terminal sequence of phosphatidylcholine on the outer part of transfersomes, inducing a higher positive ζ-potential. In this way, an increase in phosphatidylcholine content resulted in an overall decrease (absolute value) of ζ-potential.

**Figure 5:**
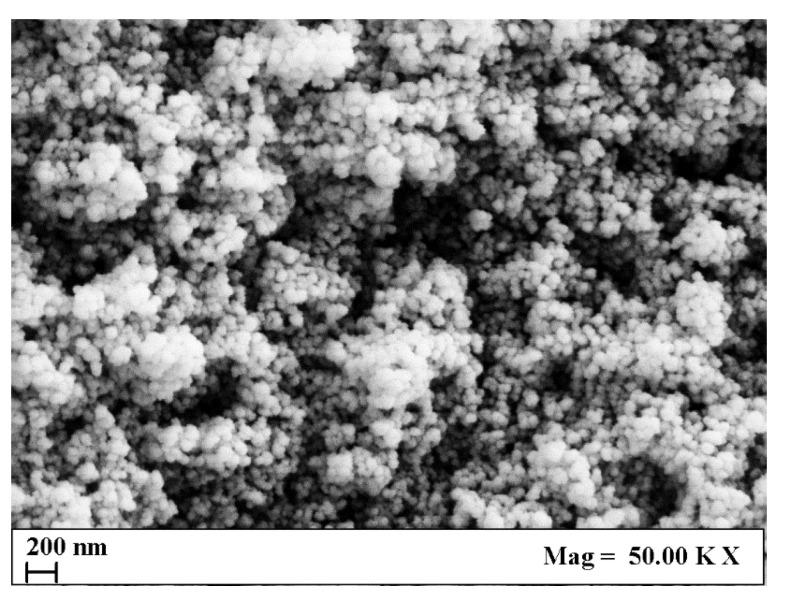
SEM image of T07 vesicles produced by SuperSomes.

Figure 5 reports an example of the nanometric and spherical morphology observed for T07 vesicles prepared at the largest phosphatidylcholine amount (3000 mg) tested in this work.

### 3.3. Production of Transfersomes Loaded with Ascorbic Acid

In the last set of experiments, transfersomes loaded with ascorbic acid were prepared. The previous experiments showed that Span80 was the right candidate as an edge activator and 80:20 was the optimal phosphatidylcholine to edge activator weight ratio to produce vesicles stable for a longer time. Therefore, ascorbic acid loaded transfersomes were produced by SuperSomes using Span80, a phosphatidylcholine to edge activator weight ratio equal to 80:20, and the two different phosphatidylcholine amounts previously analysed (2000 and 3000 mg), to study the deformability of the vesicles, and the encapsulation efficiency, release rate and antioxidant activity of the drug. The tests performed in this set of experiments are named T10 and T11.

Nanometric transfersomes were also produced in this case, with a mean diameter of 153 and 185 nm for T10 and T11, respectively (see Table 2). Both samples loaded with ascorbic acid showed an increase in MHD of about 12% with respect to the one measured for the corresponding empty samples (T06 and T09). ζ-potential values of the two formulations were comparable (−26.2 for T10 and −25.1 for T11) and did not change significantly with respect to empty transfersomes.

**Figure 6:**
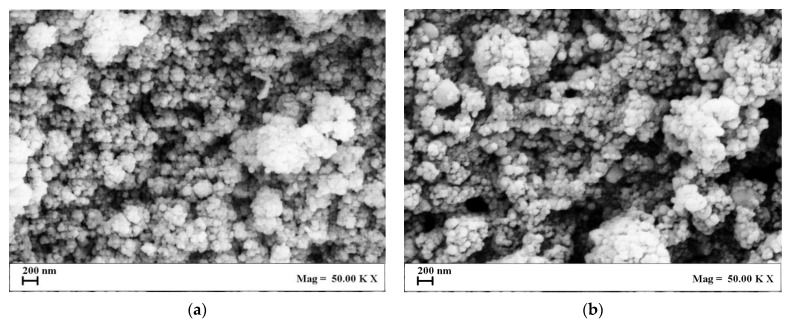
SEM images of (**a**) T10 and (**b**) T11 vesicles, produced by SuperSomes.

SEM images are reported in Figure 6a,b and show that, also in the case of drug loaded transfersomes, spherical nanometric vesicles were obtained.

Ascorbic acid encapsulation efficiency was 94% for T10 samples and 96% for T11 samples. These comparable values indicated that the phosphatidylcholine amount had no a significant effect on the drug encapsulation efficiency since, in both cases, it was in large excess with respect to the drug and the entrapment mechanism during the supercritical process due to the formation of a first layer of lipids around the water droplets was fast enough to avoid drug leakage.

**Figure 7:**
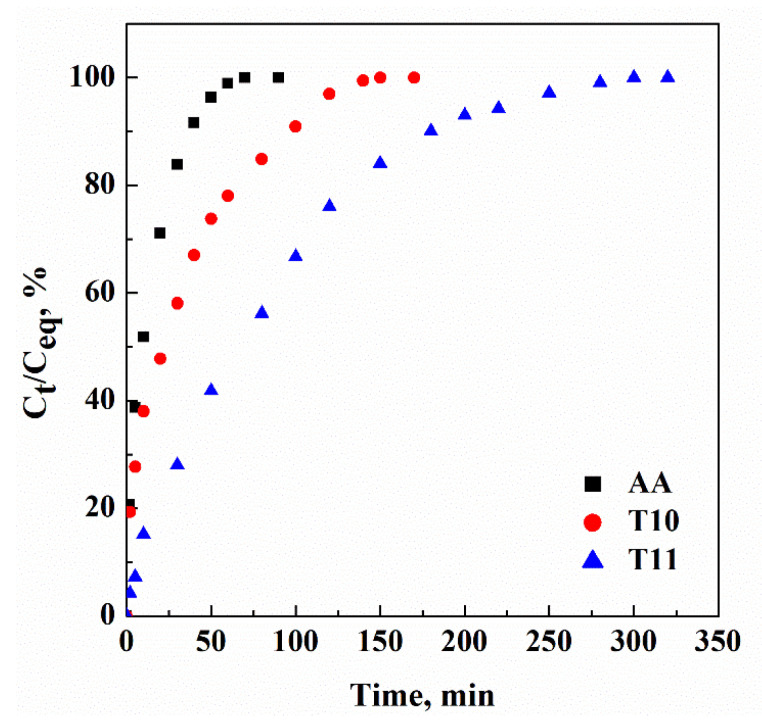
Release profiles of ascorbic acid alone (AA) and ascorbic acid loaded in T10 and T11 transfersomes, produced by SuperSomes.

The measurement of the release rate of ascorbic acid from transfersomes was carried out following the method described in Section 2.3 and drug release profiles are reported in Figure 7 as the ratio between the drug concentration measured at specific time intervals (C_t_) and the maximum drug concentration released from the vesicles (C_eq_) against time.

The results show that ascorbic acid alone was completely dissolved in the PBS medium in about 70 min; whereas its release from transfersomes was prolonged. In particular, T10 and T11 samples showed an ascorbic acid release time of approximately 150 and 300 min, respectively. Therefore, transfersomes produced using 3000 mg phosphatidylcholine (T11) had a doubled release time of ascorbic acid compared to transfersomes produced using 2000 mg phosphatidylcholine (T10). This result was due to the larger amount of phosphatidylcholine, which led to a decrease in the permeability of the vesicle bilayer and, consequently, promoted a slower drug release rate [7].

For this reason, T11 transfersomes were selected as a model sample to perform the deformability test. According to the procedure described in Section 2.3, the aqueous suspension of loaded transfersomes was forced to pass through a 0.1 µm porous membrane using a vacuum pump. At the end of the test, the collected sample was analysed by DLS, and the largest transfersomes diameter read on the size distribution curve was equal to 220 nm. Therefore, the deformability factor, obtained by the ratio between this diameter (220 nm) and the pore size of the membrane (100 nm), was equal to 2.2.

**Figure 8:**
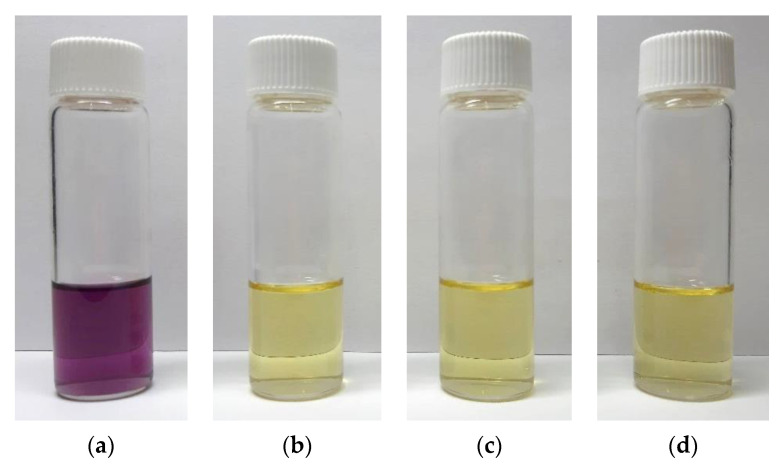
Color change of DPPH ethanol solution: (**a**) Starting DPPH solution, (**b**) DPPH after the contact with untreated ascorbic acid powder, (**c**) DPPH after the contact with T10 transfersomes, (**d**) DPPH after the contact with T11 transfersomes.

The antioxidant activity of the ascorbic acid encapsulated in transfersomes was investigated using the DPPH method. The analysis was performed on T10 and T11 samples, and was compared with the results of an aqueous solution of ascorbic acid at the same drug concentration present in the transfersomes. Figure 8 shows the color of the ethanol solution of DPPH before (Figure 8a) and after its contact with the antioxidant drug in form of untreated powder (Figure 8b) and encapsulated in transfersomes (see T10 in Figure 8c and T11 in Figure 8d).

In all cases, the DPPH solution, after contact with ascorbic acid, changed from purple to yellow, indicating, from a qualitative viewpoint, that the antioxidant action of ascorbic acid was preserved after supercritical processing. The value of DPPH radical scavenging activity confirmed this observation: i.e., it was equal to 94% for untreated ascorbic acid powder and 93% for both T10 and T11 samples, as a consequence of the high drug encapsulation efficiency (94% and 96%, respectively).

## 4. Conclusions

In this work, empty and ascorbic acid loaded transfersomes were successfully produced, by using, for the first time, a supercritical CO_2_ assisted process. Span80 was selected as the proper edge activator that, coupled with the largest phosphatidylcholine amount (3000 mg) tested in this work, produced a longer drug release time of up to 5 h. The lipid quantity slightly affected the ascorbic acid encapsulation efficiency that reached 96%, determining a quasi-100% DPPH radical scavenging activity of the loaded transfersomes. These results demonstrated the efficacy of the supercritical process to produce loaded deformable nano-transfersomes that preserved the activity of the encapsulated drug.

Future studies will be focused on the in vivo validation of these nano-vesicles for transdermal drug delivery in order to ensure quality, safety and efficacy of such products.

## Figures and Tables

**Table 1:** Formulations tested for transfersomes production by SuperSomes.

Formulation	Phosphatidylcholine Amount, mg	Edge Activator	Hydrophilic to Lipophilic Balance (HLB)	Phosphatidylcholine to Edge Activator Ratio, mg/mg	Drug Concentration in Water, mg/mL
T01	2000	Tween80	15	95:5	-
T02	2000	Tween80	15	90:10	-
T03	2000	Tween80	15	80:20	-
T04	2000	Span80	4.3	95:5	-
T05	2000	Span80	4.3	90:10	-
T06	2000	Span80	4.3	80:20	-
T07	3000	Span80	4.3	95:5	-
T08	3000	Span80	4.3	90:10	-
T09	3000	Span80	4.3	80:20	-
T10	2000	Span80	4.3	80:20	0.5
T11	3000	Span80	4.3	80:20	0.5

**Table 2:** DLS results related to transfersomes produced in this work by SuperSomes.

Formulation	MHD, nm	PDI	ζ-Potential, mV
T01	106 ± 30	0.284	−14.1 ± 1.9
T02	111 ± 40	0.267	−21.4 ± 2.9
T03	130 ± 57	0.361	−27.1 ± 3.3
T04	105 ± 30	0.281	−19.4 ± 2.7
T05	118 ± 48	0.243	−23.9 ± 3.9
T06	138 ± 55	0.279	−30.4 ± 2.4
T07	126 ± 50	0.337	−16.9 ± 2.3
T08	143 ± 56	0.327	−17.2 ± 1.9
T09	164 ± 51	0.351	−26.2 ± 2.4
T10	153 ± 71	0.404	−26.2 ± 2.5
T11	185 ± 46	0.349	−25.1 ± 1.8

**Table 3:** Stability measurements over time of the samples related to the experiments from T01 to T06.

Formulation	Stability, Days	MHD, nm	PDI	ζ-Potential, mV
T01	0	106 ± 30	0.284	−14.1 ± 1.9
	15	107 ± 40	0.333	−14.5 ± 3.8
	30	104 ± 54	0.405	−10.6 ± 2.1
	90	43 ± 10 175 ± 19	Bimodal	−11.5 ± 1.2
T02	0	111 ± 40	0.267	−21.4 ± 2.9
	15	109 ± 26	0.349	−21.6 ± 2.1
	30	110 ± 51	0.376	−21.3 ± 2.0
	90	103 ± 51	0.518	−17.9 ± 2.2
T03	0	130 ± 57	0.361	−27.1 ± 3.3
	15	72 ± 12 435 ± 70	Bimodal	−27.9 ± 2.2
	30	76 ± 11 447 ± 58	Bimodal	−16.7 ± 2.5
	90	110 ± 21 545 ± 90	Bimodal	−19.9 ± 2.8
T04	0	105 ± 30	0.281	−19.4 ± 2.7
	15	91 ± 33	0.272	−21.1 ± 2.7
	30	91 ± 37	0.367	−18.1 ± 1.6
	90	100 ± 39	0.445	−15.9 ± 2.9
T05	0	118 ± 48	0.243	−23.9 ± 3.9
	15	119 ± 42	0.275	−23.3 ± 3.4
	30	117 ± 51	0.254	−22.4 ± 3.3
	90	105 ± 41	0.265	−19.8 ± 3.7
T06	0	138 ± 55	0.279	−30.4 ± 2.4
	15	137 ± 65	0.295	−28.0 ± 2.7
	30	138 ± 46	0.272	−30.2 ± 2.8
	90	138 ± 56	0.378	−26.0 ± 2.0

## Data Availability

Data sharing not applicable.
